# Cancer Specific Mortality in Insulin-Treated Type 2 Diabetes Patients

**DOI:** 10.1371/journal.pone.0093132

**Published:** 2014-03-25

**Authors:** Sorin Ioacara, Cristian Guja, Constantin Ionescu-Tirgoviste, Simona Fica, Michael Roden

**Affiliations:** 1 “Elias” Hospital, Bucharest, Romania; 2 “Carol Davila” University of Medicine and Pharmacy, Bucharest, Romania; 3 “N. Paulescu” National Institute of Diabetes, Nutrition and Metabolic Diseases, Bucharest, Romania; 4 Department of Endocrinology and Diabetology, Heinrich-Heine University, Düsseldorf, Germany; 5 Institute for Clinical Diabetology, German Diabetes Center, Düsseldorf, Germany; Kagoshima University Graduate School of Medical and Dental Sciences, Japan

## Abstract

**Aims:**

To test the hypothesis that cumulative exposure to insulin and long-acting insulin analogs might be associated with cancer mortality in diabetes patients.

**Methods:**

All consecutive diabetes patients aged over 40 years, residing in a major urban area were screened at their first diabetes outpatient visit between 01/01/2001-12/31/2008 (n = 79869). Exclusion criteria were insulin treatment at screening, no insulin treatment until 12/31/2008, less than 6 months of glucose-lowering treatment alone before insulin initiation, insulin prescription before glargine became available, age <40/≥80 years at first insulin prescription, and <6 months of insulin exposure. A total 4990 subjects were followed-up for death based on death certificate, until 12/31/2011. Adjusted time-dependent competing risk regression analysis, with daily updates of treatment modalities was performed. Results are expressed for every 10,000 IU of cumulative dose or one year of cumulative time exposure to insulin.

**Results:**

Mean baseline age was 62±9 years, and follow-up 4.7±1.9 years. Glargine cumulative dose was associated with lower cancer mortality risk (subhazard ratio, SHR: 0.94 (95%CI 0.89–0.99, p = 0.033)). Cumulative exposure limited to that attained one year prior to death revealed lower SHRs for cumulative time (0.94 (95%CI 0.89–0.99, p = 0.018)) and cumulative dose of glargine (0.92 (95%CI 0.86–0.98, p = 0.014)). Glargine cumulative time and cumulative dose were significant predictors for lower pancreatic and breast cancer mortality, but not for deaths from lung, colorectal, female genital, liver, and urinary tract cancer. No increased hazards were found for any other subtypes of insulins.

**Conclusions:**

The cumulative dose exposure to insulin glargine was associated with a lower risk of cancer mortality in general, and of breast and pancreatic cancer in particular. This effect remained even after additional “fixed” cohort or propensity score analyses.

## Introduction

Insulin deficiency is the key abnormality in both type 1 and type 2 diabetes. Most type 2 diabetes patients eventually require insulin treatment if survival is long enough. The need to achieve appropriate insulin concentrations in the fasted state led to the development of so-called basal insulins or long-acting insulin analogs. While the currently available basal insulin analogs, glargine and detemir, offer improved pharmacokinetic properties, previous surveys reported conflicting data on the incidence of cancer at least during high-dose treatment with insulin glargine [1]–[3]. These findings received broad attention, because even human insulin is able to stimulate pathways which contribute to growth and cellular proliferation [4]. Recently, the ORIGIN trial found no evidence for increased cancer incidence or mortality in patients with impaired glucose metabolism or early type 2 diabetes [5]. However, this study employed patients in early phases of diabetes, a significant number of them being classified in the pre-diabetes range. It still remains unclear whether initiating insulin in patients with a longer history of oral drug therapy alone does associates with increased cancer incidence and cancer mortality.

The aim of the study was to investigate the hypothesis that cumulative exposure to insulin and long-acting insulin analogs in previously oral treated type 2 diabetes patients might be associated with increased cancer mortality.

## Methods

### Study Population

The study was approved by the “N. Paulescu” National Institute of Diabetes, Nutrition and Metabolic diseases ethic committee. Informed consent was not obtained from study participants as this is a purely observational, non-interventional, retrospective study of local standards of care. This is a database driven study, based on an anonymized version of the “N. Paulescu” mortality database. Study design was discussed with the Ethic Committee, and no waiver was released for the lack of informed consent because this is the local standard procedure for this type of database driven study, and not an exception. A second paper was released acknowledging the approval for use of the database.

As part of the larger Bucharest Diabetes Mortality study, all consecutive diabetes patients receiving an oral glucose lowering drug (OGLD) or insulin prescription from two major regional outpatient clinics from January 1, 2001 to December 31, 2008 were screened [6]. During the first six years of inclusion period, these two centres were the only sites distributing OGLD and insulin for free in the Bucharest region (Romania). This ensures the highest possible access to the diabetes population in the area. The date of screening was considered the date of first diabetes prescription. Screened subjects were a mix of individuals with newly discovered and previous diagnosed diabetes, attending their regular diabetes consultation.

**Figure 1:**
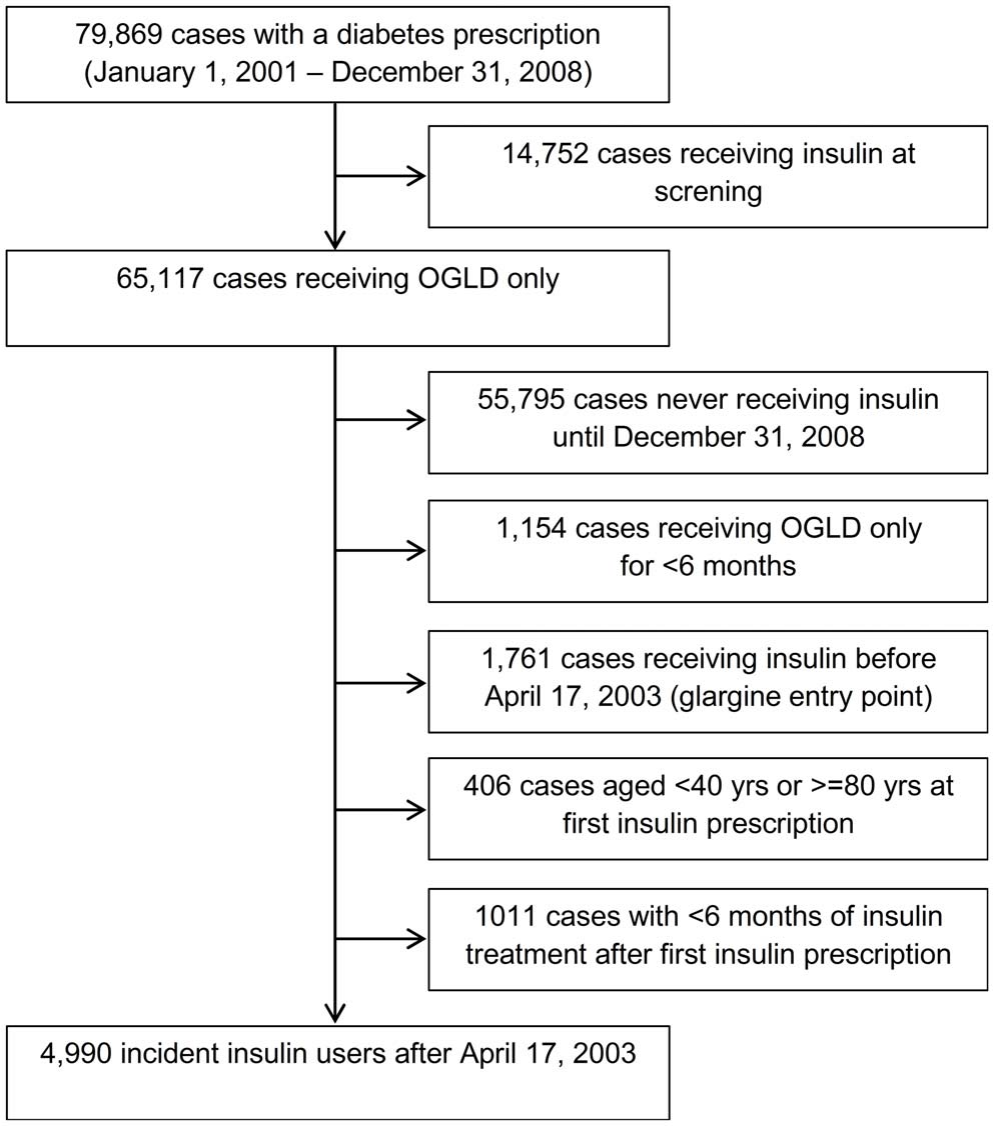
Flow chart of patient recruitment.

Patients’ recruitment is described in Figure 1. Patients receiving insulin before April 17, 2003 were excluded because long acting insulin analogs were not available before that date. Patients receiving OGLD for less than 6 months were also excluded in order to obtain a pool of truly OGLD-treated patients. A minimum of 6 months of follow-up (and insulin treatment) after the first insulin prescription was considered mandatory to avoid the protopathic bias. The final study cohort retained diabetes cases of incident insulin users, having their first insulin prescription after glargine was available. There were no special prescription indications for any insulin type, including glargine. All insulins were available for free. Although all possible precautions were taken in order to ensure a cohort of type 2 diabetes patients, inclusion of some type 1 diabetes cases, perhaps latent autoimmune diabetes in adults couldn’t be completely avoided.

Inception point was defined at six months after the first insulin prescription. All patients were followed-up for vital status until December 31, 2011 by cross linking with the national mortality database. All data are retrospective. Causes of death were based on the death certificate data, using the International Statistical Classification of Diseases and Related Health Problems 10^th^ Revision (ICD-10; www.who.int/classifications/icd/en/).

### Exposure Time

The various diabetes treatment options were coded according to the following non-mutually exclusive classes: regular (rapid-acting) insulin, rapid-acting analogs, premixed human insulin, premixed analogue insulin, long and intermediate human insulin - mostly Neutral Protamine Hagedorn (NPH), glargine, detemir, metformin, glimepiride, gliclazide-MR, glipizide, glibenclamide, repaglinide, pioglitazone and rosiglitazone. For each patient, the cumulative number of days and cumulative dose were calculated for each treatment option. Diabetes prescriptions were not available after December 31, 2008 so that the last observation was carried forward until death or end of follow-up on December 31, 2011. This significantly reduces the reverse causation introduced by a change in treatment due to an (undiagnosed) cancer, around the end of follow-up. We acknowledge the trade-off for some treatment misclassification during this period.

### Outcomes

The primary outcome was cancer death based on ICD-10 codes ranging C00-D09. Secondary outcome was site-specific cancer death based on ICD-10 codes. Sites with at least ten registered deaths were evaluated: colorectal (C18-C20), liver (C22), pancreatic (C25), lung (C34), breast (C50), female genital (C51-C59), and urinary tract (C64-C68).

### Confounders

The time between date of screening and date of first insulin prescription was used to at least partially adjust for the unavailable disease duration. The defined daily dose represents the average prescribed daily dose for a certain drug [7]. The dose of each OGLD taken before insulin initiation was divided by the corresponding defined daily dose of each drug. The sum of all these results generates a new variable, called here treatment intensity level (TIL), as a measure of disease severity. Age at cohort inception and gender were included as possible confounders. As our focus is directed towards insulin associated hazards, it is worth remembering that results are also adjusted for the concomitant use of various oral diabetes medications.

### Statistical Analysis

Statistical analysis used parametric and nonparametric tests according to the data set. Chi-square test was used for assessing differences in percentages or rates. Statistical relevance was considered for p<0.05. Crude mortality rates (CMR) were calculated per 1000 person years. The relationship between insulin use and cancer mortality was analyzed using the competing-risks regression by the method of Fine and Gray [8]. Competing risk events prevent the outcome of interest from occurring, as opposed to censoring, which prevent it from being observed. In the presence of significant competing events, standard Cox regression analysis is less reliable in estimating hazards associated with covariates of interest. In this study, all other deaths than the defined outcome were considered competing events. The competing-risks analysis results are subhazard ratios (SHR), which are interpreted similar to hazard ratios from the classic Cox regression analysis. The exposure, dose, cumulative exposure, and cumulative dose to all treatment modalities were available as time-dependent variables. The competing risk regression model simultaneously used both cumulative exposure and the categorical ever exposed variable describing the available treatment options. This method has been developed to address the inherent ‘frailty’ of the data resulting from the lack of randomization [9]. It was previously demonstrated that inclusion of time-dependent ever exposure, as done here, leads to risk estimates for cumulative variables that are not influenced by the allocation bias [9]. The ever exposed results are not of interest, as they entered the model only for adjustment reasons [9].

The risk associated with cumulative dose was calculated for every 10,000 increment in corresponding standard units of measurement, i.e. for every 10,000 IU cumulative dose of any insulin preparation. Detemir exposure was not taken into account because it was not available at baseline, but only after May 2007. It can be algebraically shown that the Kaplan-Meier method constantly provides overestimated results in the presence of competing events. The true estimates can be calculated and visually displayed using the competing risk cumulative incidence functions, as used in this study. The competing-risks statistical model was similarly constructed when all cancer or site-specific cancer deaths were used as defined outcome. Besides the “as treated” analysis, a “fixed-cohort” analysis was performed using the first insulin category prescribed, which then was “fixed” (i.e. not time-dependent) for the entire follow-up. Furthermore, analysis of cumulative exposure up until one year before death or end of follow-up was performed to once again address the potential reverse causation bias. Propensity-score analysis was performed to minimize the residual confounding bias. Propensity scores were calculated from the baseline variable values. Finally, standard time dependant Cox regression analysis was performed to assess the impact of competing events on results. All calculations were performed using STATA 13 software (www.stata.com).

## Results

### Comparison of Ever and Never Glargine Users

Applying the prespecified exclusion criteria resulted in 4990 cases (41.8% males) of incident insulin users among 79869 individuals initially screened at their first diabetes prescription between January 1, 2001 and December 31, 2008 (Figure 1). Users and non-users of insulin glargine differed at baseline and during follow-up. At baseline, patients ever exposed to glargine were younger and more likely to receive an OGLD combined with insulin compared with never glargine users (Table 1). During follow-up, the average daily insulin dose and duration of follow-up were slightly lower in ever users compared with never users of glargine insulin. There were no significant differences in gender distribution and screening to first insulin prescription time lag.

**Table 1:** Characteristics of ever and never glargine users.

	Ever glargine users	Never glargine users
n (%)	1425 (28.6%)	3565 (71.4%)
Males n (%)^a^	595 (41.8%)	1483 (41.6%)^NS^
Age at inception (years)	61.7±9.3	62.3±9.3*
Baseline use of OGLD n (%)^a^		
Sulfonylureas	530 (37.2%)	608 (17.1%)
Repaglinide	100 (7%)	58 (1.6%)
Metformin	459 (32.2%)	724 (20.3%)
Pioglitazone	43 (3.0%)	83 (2.3%)
Rosiglitazone	21 (1.5%)	35 (1.0%)
TIL	1.50±0.54	1.45±0.56*
Time between screening and FIP (years)	4.0±1.9	3.9±1.9^NS^
Follow-up (years)	4.8±1.8	4.6±1.9**
Daily dose of insulin during follow-up (U)	33±14	37±14***

a Percent within ever and never users, respectively.

OGLD oral glucose lowering drugs,TIL treatment intensity level (see Methods), FIP first insulin prescription.

NS not significant, *p<0.05, **p<0.01, ***p<0.001 vs. ever glargine users.

### As-treated Analysis

The mean follow-up was 4.65±1.88 years. During 23179 person-years exposure time, 887 died (CMR 38.27/1000 person-years). There were 160 deaths (18% of total deaths, CMR 6.90/1000 person-years) from cancer and 727 deaths from non-cancer causes, including 521 cardiovascular deaths. Cumulative exposure time to glargine insulin was associated with a trend towards lower cancer mortality (SHR 0.96, 95% CI 0.92–1.01, p = 0.091), without reaching statistical significance (Table 2). There was a significant reduction in cancer mortality (sub)hazard for cumulative time exposure to premixed human (SHR 0.945, 95% CI 0.918–0.971, p = 0.001) and analogue insulins (SHR 0.962, 95% CI 0.935–0.990, p = 0.009), and for cumulative dose exposure to premixed human insulin (SHR 0.955, 95% CI 0.926–0.985, p = 0.004). No significant impact on cancer mortality was registered for human basal, regular or rapid-acting analogs as regards both cumulative time and cumulative dose exposure.

**Table 2:** Competing risk analysis of cancer specific mortality.

	Cumulative time		Cumulative dose	
Covariables	SHR (CI95%)^a^	p	SHR (CI95%)	p
Cancer deaths (n)	160	–	160	–
Competing events (n)	727	–	727	–
Age at baseline	1.033 (1.016–1.051)	0.001	1.032 (1.015–1.050)	0.001
Male gender	1.609 (1.179–2.196)	0.003	1.584 (1.160–2.164)	0.004
TIL^b^	1.295 (1.001–1.675)	0.049	1.326 (1.028–1.710)	0.030
Screening time^c^	0.888 (0.810–0.974)	0.012	0.896 (0.817–0.982)	0.019
Glargine	0.960 (0.916–1.007)	0.091	0.944 (0.894–0.995)	0.033
Basal human insulin	0.985 (0.948–1.024)	0.450	0.997 (0.953–1.043)	0.886
Regular insulin	0.999 (0.973–1.026)	0.962	0.988 (0.970–1.005)	0.168
Rapid-acting analogs	0.921 (0.822–1.033)	0.162	0.930 (0.820–1.056)	0.262
Premixed human insulin	0.945 (0.918–0.971)	0.001	0.955 (0.926–0.985)	0.004
Premixed analogue insulin	0.962 (0.935–0.990)	0.009	0.984 (0.964–1.005)	0.146
Metformin	0.954 (0.902–1.009)	0.102	0.999 (0.999–1.000)	0.184
Glimepiride	0.970 (0.919–1.023)	0.261	0.789 (0.511–1.219)	0.286
Gliclazide	0.833 (0.616–1.125)	0.233	0.963 (0.891–1.042)	0.349
Glipizide	0.971 (0.899–1.049)	0.458	0.937 (0.832–1.056)	0.287
Glibenclamide	0.993 (0.925–1.066)	0.846	0.955 (0.792–1.249)	0.966
Repaglinide	1.017 (0.966–1.070)	0.531	0.993 (0.759–1.299)	0.957
Pioglitazone	0.869 (0.763–0.990)	0.035	0.924 (0.848–1.007)	0.071
Rosiglitazone	0.954 (0.862–1.056)	0.368	0.700 (0.502–0.975)	0.035

a SHR subhazard ratios, similar to hazard ratios (HR) from the classic Cox regression.

b Treatment intensity level (see Methods).

c Time between screening and date of insulin initiation(years).

**Figure 2:**
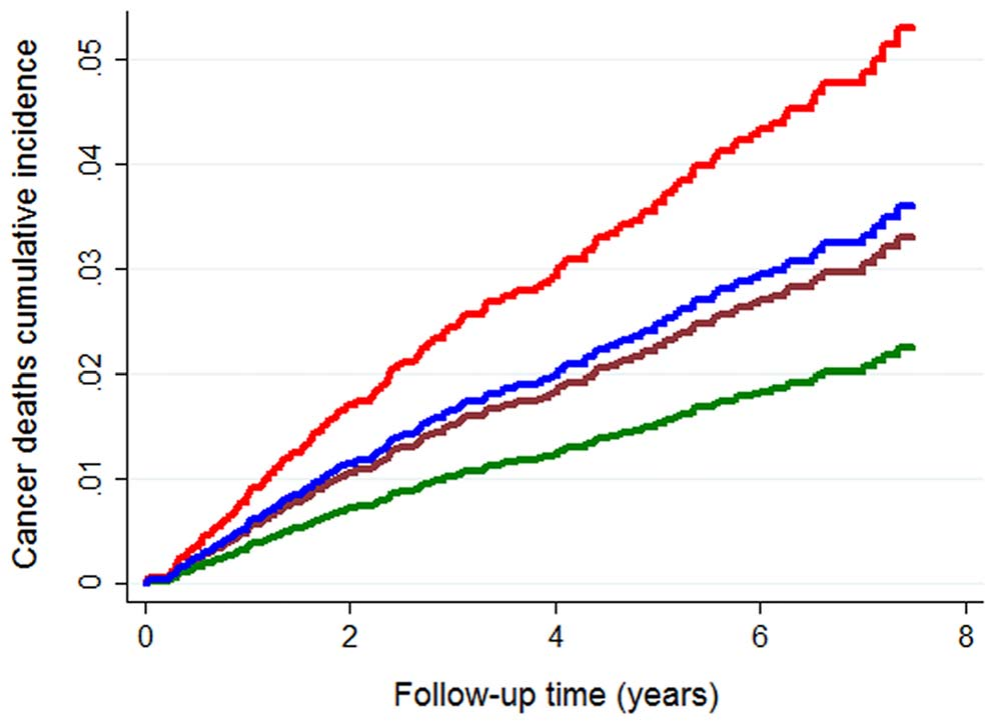
Cancer deaths cumulative incidence functions. Green line, females exposed to glargine; brown line, females unexposed to glargine; blue lines, males exposed to glargine; red line, males unexposed to glargine.

The cumulative dose assessment of exposure to glargine insulin revealed a modest protective effect for cancer mortality (SHR 0.94, 95% CI 0.89–0.99, p = 0.033). Figure 2 shows the cumulative incidence functions for cancer death by gender and follow-up exposure to glargine. If the cumulative exposure was limited to that attained one year prior to death (minimizing the reverse causation), all point estimates were similar, i.e. glargine cumulative exposure time SHR 0.938 (95% CI 0.889–0.989, p = 0.018) and cumulative dose SHR 0.919 (95% CI 0.859–0.983, p = 0.014).

The main sites of cancer deaths (n, % total cancers) were: lung (n = 36, 22.5%), colorectal (n = 20, 12.5%), female genital (n = 18, 11.2%), liver (n = 15, 9.4%), pancreatic (n = 15, 9.4%), breast (n = 13, 8.1%), urinary tract (n = 11, 6.9%), and others (n = 32, 20.0%). Using the same full model as for all cancers, glargine use was associated with cumulative exposure time and cumulative dose that were significant predictors for lower pancreatic and breast cancer mortality, with no significant impact on lung, colorectal, female genital, liver, and urinary tract cancer deaths (Table 3).

**Table 3:** Competing risk analysis of site-specific cancer mortality for glargine exposure.

	Cumulative exposure		Cumulative dose	
Cancer site^a^	SHR (CI 95%)^b^	p	SHR (CI 95%)	p
Lung (n = 36)	1.008 (0.965–1.054)	0.723	0.964 (0.897–1.036)	0.319
Colorectal (n = 20)	0.887 (0.657–1.197)	0.433	0.913 (0.729–1.144)	0.428
Female genital (n = 18)	1.022 (0.895–1.167)	0.749	0.956 (0.823–1.110)	0.551
Liver (n = 15)	0.985 (0.906–1.072)	0.733	1.022 (0.950–1.099)	0.562
Pancreatic (n = 15)	5*10^−6^ (6*10^−9^–0.005)	<0.05^c^	9*10^−5^ (1*10^−7^–0.058)	0.005
Breast (n = 13)	0.762 (0.663–0.877)	0.001	0.849 (0.769–0.937)	0.001
Urinary tract (n = 11)	0.885 (0.761–1.030)	0.114	0.933 (0.828–1.051)	0.252

a Although not shown here, the model also included age at first insulin prescription (FIP), gender, time between screening and FIP, treatment intensity level and all other available oral or insulin categories as both cumulative exposure and time-dependent ever exposed terms (see Methods).

b SHR, subhazard ratios, similar to hazard ratios from the classic Cox regression.

c Significant result rounded up to <0.05 due to very low estimated SHR.

### Fixed-cohort Analyses and Propensity-score Analyses

Propensity score analysis did not changed the previously calculated hazards by more than 5%.

When the time-dependent nature of treatment was ignored and predictors’ levels were “fixed” at their baseline values, the point estimates for insulin initiation with insulin glargine were: all cancers SHR 0.67 (95% CI 0.31–1.48, p = 0.326), lung cancer SHR 0.52 (95% CI 0.10–2.71, p = 0.439), colorectal cancer SHR 2.9 (95% CI 0.50–16.82, p = 0.233), female genital cancer SHR 0.23 (95% CI 0.04–1.42, p = 0.114), liver cancer SHR 0.23 (95% CI 0.01–3.98, p = 0.312), pancreatic cancer SHR 0.40 (95% CI 0.03–6.11, p = 0.511), breast cancer SHR 1.02 (95% CI 0.14–7.72, p = 0.980), and urinary tract cancer SHR 0.47 (95% CI 0.07–3.23, p = 0.440).

### Assessment of Competing Risk Impact on Results

Removing all adjustments made for competing risks by using the time dependent Cox regression analysis yielded the following all cancers hazards for cumulative dose: glargine 0.903 (95% CI 0.849–0.962, p = 0.002), human premixed insulin 0.925 (95% CI 0.899–0.952, p<0.001), premixed analogue insulin 0.955 (95% CI 0.931–0.979, p<0.001), and regular insulin 0.966 (95% CI 0.936–0.997, p = 0.03), with no significant impact for any other treatment option. Similarly, cumulative time exposure hazards were: human premixed insulin 0.886 (95% CI 0.856–0.917, p<0.001), premixed analogue insulin 0.899 (95% CI 0.867–0.932, p<0.001), glargine 0.905 (95% CI 0.859–0.954, p<0.001), human basal insulin 0.944 (95% CI 0.900–0.991, p = 0.019), regular insulin 0.954 (95% CI 0.915–0.994, p = 0.024), pioglitazone 0.854 (95% CI 0.743–0.982, p = 0.027), and metformin 0.948 (95% CI 0.902–0.997, p = 0.039), with no significant impact for any other treatment option.

## Conclusions

In this study, the cumulative dose exposure to insulin glargine was found to be associated with a small, but significantly lower risk of cancer in general (SHR 0.94, 95% CI 0.89–0.99, p = 0.033), and of breast and pancreatic cancer in particular. Also, the cumulative time exposure to glargine showed a trend towards lower overall cancer risk, with a significant reduction in breast and pancreatic cancer mortality. Lung, colorectal, female genital, liver and urinary tract cancer mortality were not significantly influenced by glargine exposure.

Our results regarding glargine exposure, especially on breast cancer mortality risk are contrasting some [1]–[3], [10], [11], but not all previous studies [12]–[17]. Similar to previous studies, we performed an observational, non-interventional, pharmaco-epidemiological study, acknowledging all the drawbacks that come from this approach. Discussions start with a critical approach on how the previously published recommendations for quality assurance were integrated. This ensures qualification for the so-called “second generation” studies [18]. Then, modern approaches introduced in this study are evaluated for their added value in understanding the complex relationship between diabetes and cancer.

In addition to all-type cancer, site and gender specific cancers were also evaluated. The cohort was designed to include incident users of insulin. Previous OGLD only treatment for a minimum of six months ensures a better homogeneity as regards both insulin deficiency and sensitivity among participants, also excluding almost all type 1 diabetes cases. Disease severity at insulin initiation was further evaluated by treatment intensity level, which proved to be an important predictor of cancer mortality (table 2). Some key covariates like age, gender and mean follow-up daily insulin dose were available, but many others like smoking, diabetes duration, HbA1c and body mass index were not. As expected, age and male gender were highly significant predictors of cancer mortality. Smoking and body mass index had a rather low influence on cancer mortality in previous studies [1], [3]. It is very unlikely that, in the daily practice, the decision of using one type of insulin versus another would be influenced by these two variables. Moreover, lung cancer mortality risk, which is associated with smoking status, was not increased by glargine exposure in our study. We acknowledge the potential for lifestyle related confounding, but we also estimate a minor impact on results, given the protocol restrains and the statistical approach. Previous studies, including a large randomized controlled trial found not relationship between HbA1c (glucose control) and cancer risk [19], [20]. Very short diabetes duration indirectly increases cancer risk because of detection bias, associated with increased cancer screening following diabetes diagnosis. Afterwards, diabetes duration does not add value to cancer risk assessment [21], [22]. In our study, all subjects had at least 12 months of diabetes duration because the protocol required at least 6 months of OGLD and then, another 6 months of insulin treatment. Diabetes duration was actually much higher for most individuals because the mean time between coming under observation and first insulin prescription was 4±2 years. Detection bias and reverse causality surrounding the diabetes diagnosis and insulin initiation were addressed by these protocol driven measures. Consequently, the unmeasured diabetes duration, if at all, had minimal effect on the outcomes. There is a reasonable concern that following the publication in the summer of 2009 of the four famous papers on glargine associated risk of cancer, the prescription of glargine in patients perceived at been at high cancer risk had changed in various degrees [1]–[3], [12]. In our study, all patients were included before 2009, and no new diabetes prescription was taken into account after January 1, 2009 (last observation was carried forward). In order to increase the biological plausibility of the timeframe between insulin exposure and cancer event, a separate analysis was performed using the cumulative time and dose exposure up to one year before cancer event or end of follow-up. This approach also tackles the reverse causation bias arising from an undetected cancer changing the treatment requirements. No major changes were recorded in risk estimates, besides increased statistical significance. Although reverse causation during the first six months of insulin treatment and the last year of follow-up was confidently minimized by the above protocol and data analysis measures, there still remains an uncovered window in between these two periods. The “fixed” treatment analysis, with treatment allocation “fixed” as those at baseline was performed to address this issue. It was reassuring that no potential harm was detected using this technique. Although very useful for our purpose to minimize the impact of reverse causation, this technique suffers from many flows, mostly derived from its inability to correctly measure the exposure parameters. These differences explain the dissimilarities found in point estimates between “fixed” and “as treated” analysis. However, only the latter can correctly estimate the magnitude of risk associated with the available covariates. People ever exposed to glargine differed significantly from those never exposed (table 1). Propensity score analysis was performed in order to supplementary address this issue, but no major changes in risk estimates were found. Immortal time bias was corrected using the “inception cohort” approach, including information from before first exposure to insulin, disregarding patients receiving insulin before glargine was available on the local market, and finally, using the time-varying approach in data analysis. Using time-varying variables in all possible situations also ensured the best available measurement of exposure parameters. Using cancer mortality and death certificate diagnosis is a significant drawback comparing with cancer incidence combined with histological diagnosis. Also, follow-up time is still rather limited in terms of the long-time scales for carcinogenesis. Despite all advances in cancer treatment, the local cancer mortality is still very high, mostly due to cancer diagnosis in its late stages. Consequently, there is still a very good correlation between cancer incidence and cancer mortality. Another point for consideration would be that the outcome was equally measured for each available diabetes treatment option.

Time-dependent Cox regression analysis performs poorly in the presence of a high amount of competing risk. In this study, the number of competing risk events was more than four times higher than cancer deaths (table 2). Site specific cancer mortality analysis yields 20–70 times higher competing outcomes than events of interest (table 3). Although present in all relevant studies previously published, this issue was not addressed, mainly due to computational problems arising from data complexity. In this study, time-dependent competing risk analysis was used in all calculations, ensuring the best available method for risk estimate. We assessed the impact of competing risk adjustments on results by performing a standard time dependant Cox regression analysis, with all other statistical conditions being equal. Adjustements for competing risks reduced the overall risk estimates in terms of both amplitude and statistical significance. Another significant methodological difference comparing with previous studies on cancer risk in diabetes is the concomitant use of both time-dependent ever exposed and cumulative exposure to the available treatment options. As nicely shown elsewhere on another important topic, this approach significantly increases the precision of risk estimates by effectively minimizing the residual confounding resulting from the lack of randomization [9]. Measurement precision is also enhanced by carefully subtyping both insulins and OGLD modalities. While OGLD results are to be “interpreted” mostly as adjustments, it is of great interest to note that no insulin type was associated with any possible harm as regards cancer mortality. While the issue of cancer safety of insulin use in early phases of diabetes was conclusively covered by the Origin trial, our data suggest that in long standing type 2 diabetes patients needing and receiving insulin for metabolic control, insulin exposure is associated with a significant protective effect on cancer mortality, or at least a trend towards this important outcome.

In conclusion, in this study of incident insulin users, exposure to insulin and glargine insulin in particular was not associated with any deleterious effect on overall and site specific cancer mortality. As high quality information on this topic is very much needed, but hard to obtain, we recommend using comprehensive data analysis, including time and computational consuming techniques like time-dependent competing-risks analysis, with concomitant use of both ever exposed and cumulative measurements of exposure. The debate is far from a closure, but the inverse dose-effect association for insulins and glargine insulin exposure in particular is an argument for their benign nature as regards cancer mortality risk.
